# Supplementary material for: Syntax and prejudice: ethically-charged biases of a syntax-based hate speech recognizer unveiled
Source: PeerJ Comput Sci. 2022 Feb 3;8:e859. doi: 10.7717/peerj-cs.859 (PMC9044272; doi:10.7717/peerj-cs.859)
Supplement: Supplemental Information 1 [file peerj-cs-08-859-s001.pdf]

A RANKING OF THE MOST USED HASHTAGS ON THE CAMPAIGN TRAIL BY THE DEMOCRATIC AND REPUBLICAN PARTIES

Table 6 shows the most used hashtags by the Democratic and Republican parties during the November 2020 Presidential campaign. In detail, for each party we selected the verified Twitter account of: the presidential candidate, the vice president candidate and their political party (as shown in Tab. 2 and discussed in Section 3.4.3) and extracted from each Twitter profile, the most frequent hashtags and slogans.

Political party	Hashtag
Democratic party	#Harris2020
	#KamalaHarris
	#Biden2020
	#BidenHarris2020
	#Democrats
	#VoteBlue
	#BidenHarrisToSaveAmerica
	#JoeBidenKamalaHarris2020
Republican party	#maga
	#maga2020
	#MakeAmericaGreatAgain
	#promisekept
	#promisemade
	#GOP
	#4MoreYears
	#FourMoreYears
	#Trump2020
	#VoteRed
	#TrumpPence2020

Table 6. Most used hashtags on the campaign trail by the Democratic and republican parties
