# Supplementary material for: Syntax and prejudice: ethically-charged biases of a syntax-based hate speech recognizer unveiled
Source: PeerJ Comput Sci. 2022 Feb 3;8:e859. doi: 10.7717/peerj-cs.859 (PMC9044272; doi:10.7717/peerj-cs.859)
Supplement: Supplemental Information 2 [file peerj-cs-08-859-s002.pdf]

B *US PRESIDENTIAL ELECTION CORPUS* COMPOSITION AND COMPARISON WITH THE NATIONAL EXIT POLLS

In the list below we show tweets geolocation within the *US presidential election corpus* (Sec.3.4.3) and compare them with the National Exit Polls. We only show:

1. States those constitute at least 1% of tweets in at least one of the two datasets.
2. states with a larger gap in the two datasets.

The list is in alphabetical order. Blue rows denote states won by *Democratic party* while red rows denote those won by *Republican party*.

**State**	*GOP dataset*	*DEM dataset*
Alabama	**0.45%**	0.33%
Arizona	**1.54%**	1.22%
California	13.25%	**16.71%**
Colorado	1.13%	**1.21%**
Connecticut	0.42%	**0.49%**
Florida	**10.40%**	6.47%
Georgia	1.40%	**1.96%**
Illinois	2.17%	**2.86%**
Kentucky	**0.70%**	0.65%
Maryland	0.63%	**1.17%**
Massachusetts	1.35%	**2.40%**
Michigan	0.97%	**1.46%**
Mississippi	**0.13%**	0.09%
Montana	**0.12%**	0.06%
Nebraska	**0.28%**	0.19%
Nevada	1.25%	**1.31%**
New Jersey	1.92%	**2.00%**
New York	8.50%	**10.80%**
Ohio	1.82%	**2.94%**
Oregon	**1.17%**	1.08%
Pennsylvania	2.72%	**3.13%**
South Carolina	**0.64%**	0.53%
Tennessee	**1.30%**	0.85%
Texas	**8.46%**	7.59%
Virginia	0.89%	**1.05%**
Washington	4.08%	**4.77%**
Wisconsin	0.71%	**0.81%**
Total	68.40%	74.13%

State won by ■ Democrats ■ Republicans

Table 7. Relation between percentage of tweets in the two datasets and the National Exit Pools results
