# Supplementary material for: Syntax and prejudice: ethically-charged biases of a syntax-based hate speech recognizer unveiled
Source: PeerJ Comput Sci. 2022 Feb 3;8:e859. doi: 10.7717/peerj-cs.859 (PMC9044272; doi:10.7717/peerj-cs.859)
Supplement: Supplemental Information 3 [file peerj-cs-08-859-s003.pdf]

C REASONS GIVEN TO THE *INSIDE OUT TEST*

We analyze all the reasons given by the 10 annotators who participated in the *Inside out test* (Sec.4.1). As defined within the test, each annotator was asked if they agreed or disagreed with the label given by the model. The reason was asked when the answer was negative (*No*). Let's remember that in the *Inside out test*, the available tools were: the sentence, the respective label and the syntactic tree colored according to the activation value of each node.

The following table lists the frequency of the reasons given, highlighting how the list is made up of terms used to mention: ethnic groups, people from a certain geographical area, gender and also proper names.

**Word**	**Frequency**
Black	47
African	11
Racist	10
Woman	10
Gay	8
Mexicans	8
White	6
Stuttering	6
Democrats	5
Worst	5
Israel	4
Squat	4
Trump	4
President	4
Biden	3
Weed	3
Stupid	3
Chinese	3

Table 8. Reasons and their respective frequency given in the *Inside out test*
