# Supplementary material for: Syntax and prejudice: ethically-charged biases of a syntax-based hate speech recognizer unveiled
Source: PeerJ Comput Sci. 2022 Feb 3;8:e859. doi: 10.7717/peerj-cs.859 (PMC9044272; doi:10.7717/peerj-cs.859)
Supplement: Supplemental Information 4 [file peerj-cs-08-859-s004.pdf]

D QUALITATIVE ANALYSIS: OTHER EXAMPLES

This appendix shows some new examples of the qualitative analysis (Sec.4.3).

The following images show all parse trees obtained from KERM-HATE where, for each sentence one or more words defining *the key points* of the sentence were changed. With this approach, it is easier to visualize the prejudices taken from the model.

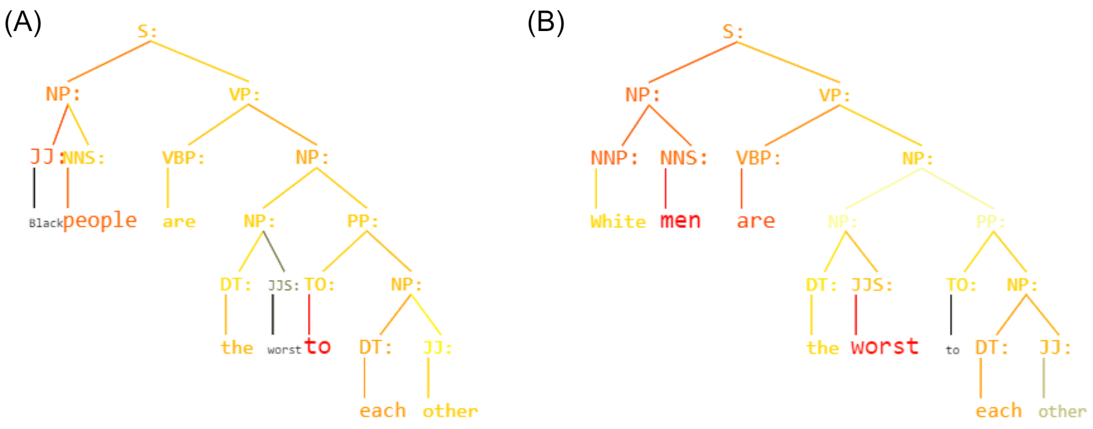

(A) Sentence: *Black people are the worst to each other* - Labeled as: **Hate speech**
(B) Sentence: *White men are the worst to each other* - Labeled as: **Neither**

Figure 5. [W] are the worst to each other

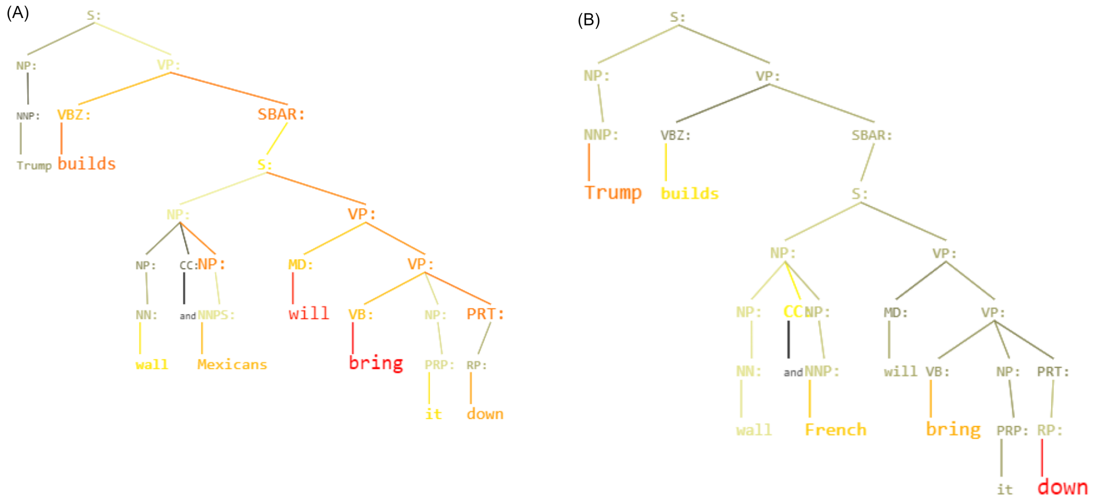

(A) Sentence: *Trump builds wall and Mexicans will bring it down* - Labeled as: **Offensive l.**
(B) Sentence: *Trump builds wall and French will bring it down* - Labeled as: **Neither**

Figure 6. Trump builds wall and [W] will bring it down

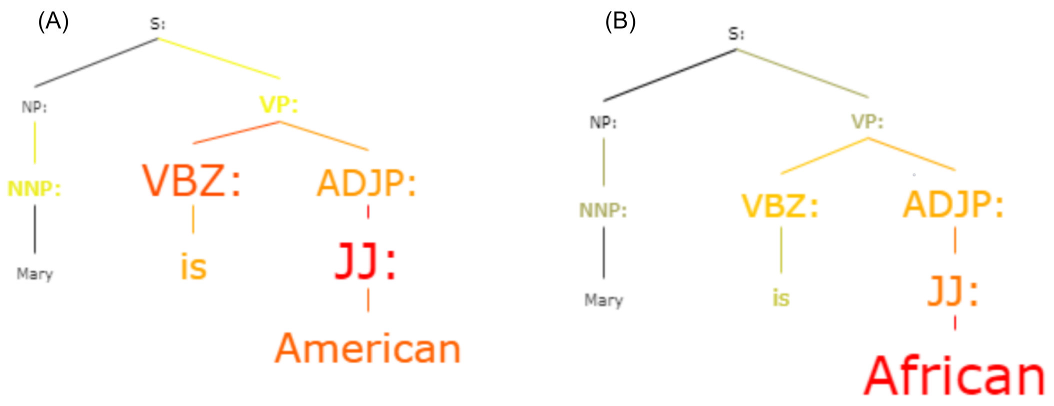

(A) Sentence: *Mary is American* - Labeled as: **Neither**
(B) Sentence: *Mary is African* - Labeled as: **Hate speech**

Figure 7. Mary is [W]
