# Supplementary figures and images for: Syntax and prejudice: ethically-charged biases of a syntax-based hate speech recognizer unveiled
Source: PeerJ Comput Sci. 2022 Feb 3;8:e859. doi: 10.7717/peerj-cs.859 (PMC9044272; doi:10.7717/peerj-cs.859)

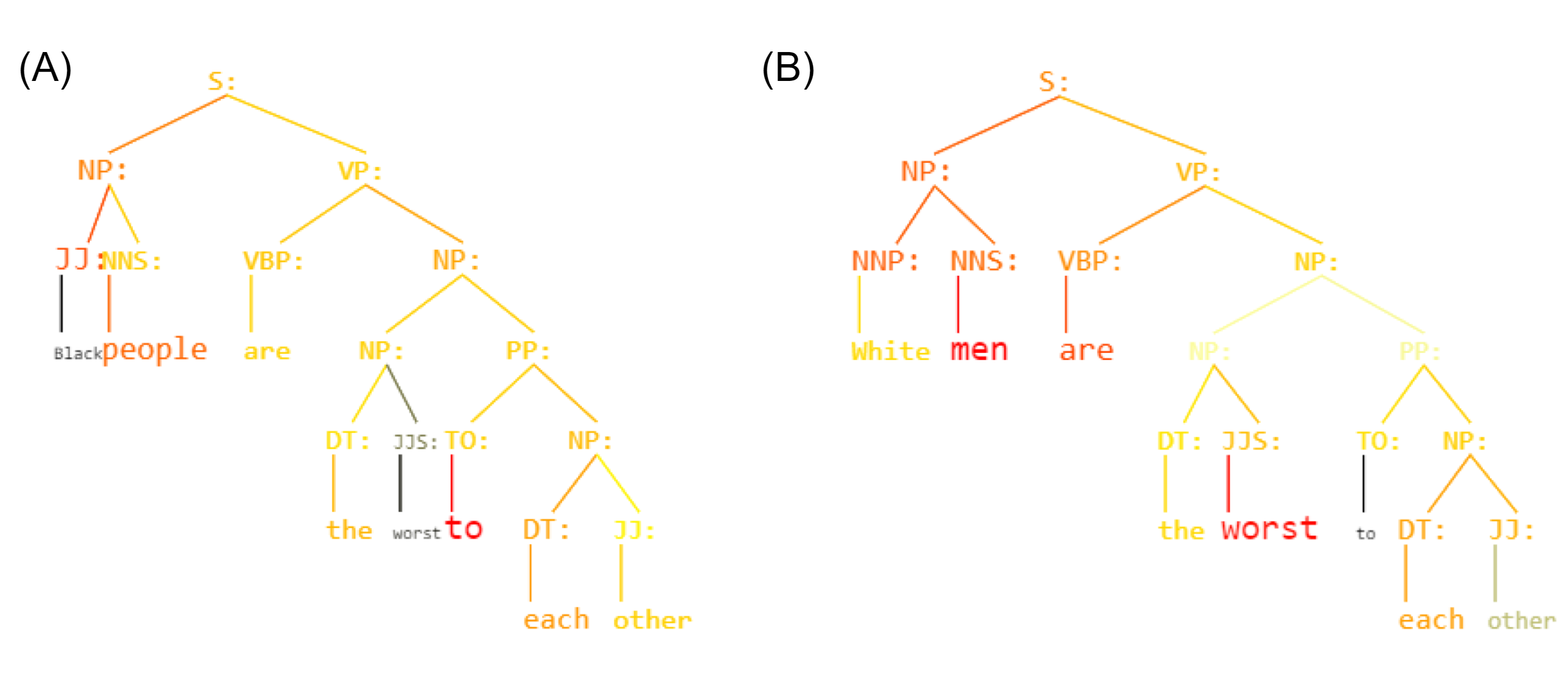

Supplement: Supplemental Information 5 [file peerj-cs-08-859-s005.png]

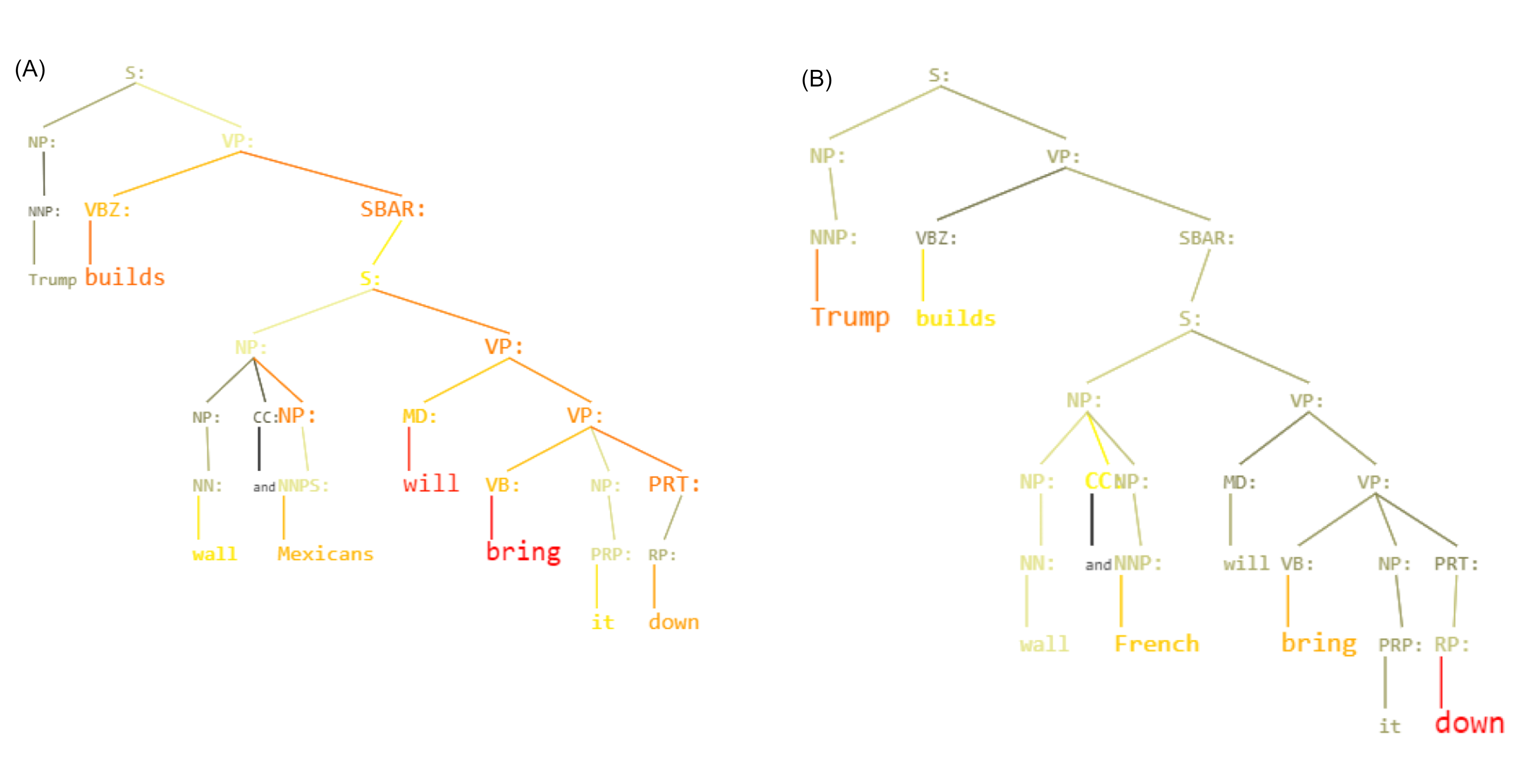

Supplement: Supplemental Information 6 [file peerj-cs-08-859-s006.png]

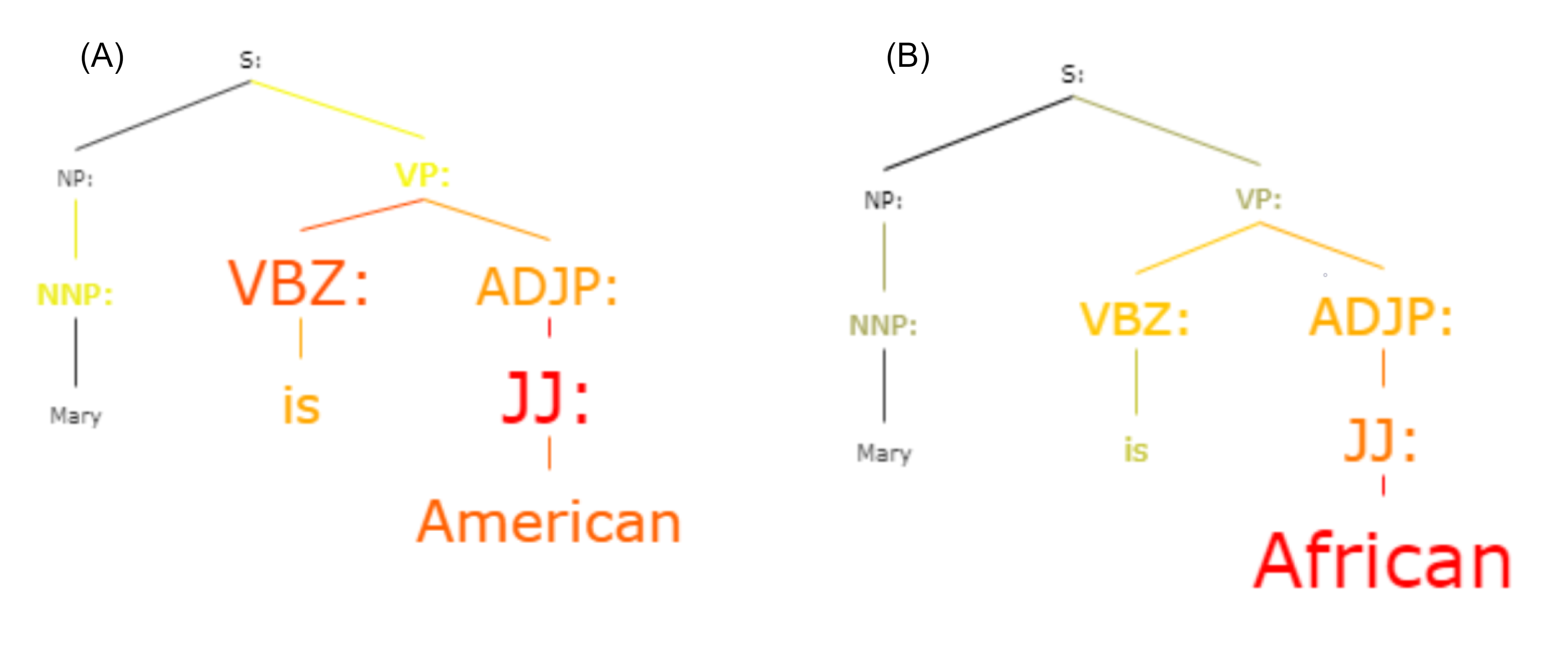

Supplement: Supplemental Information 7 [file peerj-cs-08-859-s007.png]
